# Induction of protective immune responses against schistosomiasis using functionally active cysteine peptidases

**DOI:** 10.3389/fgene.2014.00119

**Published:** 2014-05-08

**Authors:** Rashika El Ridi, Hatem Tallima, John P. Dalton, Sheila Donnelly

**Affiliations:** ^1^Zoology Department, Faculty of Science, Cairo UniversityCairo, Egypt; ^2^Medical Biology Centre, School of Biological Sciences, Queen’s University BelfastBelfast, Northern Ireland; ^3^The i-three Institute, University of Technology at SydneyUltimo, Sydney, NSW, Australia

**Keywords:** schistosome, cysteine peptidase, cathepsin B, Th2 immune response, papain

## Abstract

Each year schistosomiasis afflicts up to 600 million people in 74 tropical and sub-tropical countries, predominantly in the developing world. Yet we depend on a single drug, praziquantel, for its treatment and control. There is no vaccine available but one is urgently needed especially since praziquantel-resistant parasites are likely to emerge at some time in the future. The disease is caused by several worm species of the genus *Schistosoma*. These express several classes of papain-like cysteine peptidases, cathepsins B and L, in various tissues but particularly in their gastrodermis where they employ them as digestive enzymes. We have shown that sub-cutaneous injection of recombinant and functionally active *Schistosoma mansoni* cathepsin B1 (SmCB1), or a cathepsin L from a related parasite *Fasciola hepatica* (FhCL1), elicits highly significant protection (up to 73%) against an experimental challenge worm infection in murine models of schistosomiasis. The immune modulating properties of this subcutaneous injection can boost protection levels (up to 83%) when combined with other *S. mansoni* vaccine candidates, glyceraldehyde 3-phosphate dehydrogenase (SG3PDH) and peroxiredoxin (PRX-MAP). Here, we discuss these data in the context of the parasite’s biology and development, and provide putative mechanism by which the native-like cysteine peptidase induce protective immune responses.

Schistosomiasis is caused by several helminth (worm) species of the genus *Schistosoma*, including *Schistosoma mansoni*, *S. haematobium*, and *S. japonicum* and is endemic in 74 tropical and sub-tropical countries, most prevalently in Africa, the Middle East, South America, and South-East Asia. Diagnostic tools for the detection of infection lack sensitivity and/or specificity leading the World Health Organization (WHO) to no longer provide estimates on populations infected or at risk but rather replacing this by describing the situation as “population requiring preventive chemotherapy.” A recent WHO report (World Health Organization [WHO], 2012) revealed that the total number of people needing preventive chemotherapy globally for 2010 was over 237 million, of these > 108 million were school-age children, and of which only 13% received treatment. Praziquantel is the only readily available effective drug for the treatment of the three main parasites causing human schistosomiasis, and has the advantages of low cost and self-limiting side effects. However, complete cure is seldom achieved such that for moderate (100–400 eggs per gram feces, epg) and heavy ( >400 epg) infections the cure rate may not exceed 60%. Consequently, a substantial proportion of treated individuals can remain infected (probably unaware) and, therefore, are at risk of the serious sequelae of chronic schistosomiasis (Barsoum et al., 2013).

We cannot remain dependent on a single drug for the treatment and control of schistosomiasis as it is likely that in some time praziquantel-resistant parasites will emerge and, therefore, a vaccine for schistosomiasis is urgently needed. Schistosomes live intravascularly throughout their time spent in the mammalian host; the mature adult worms can reside in the mesenteric or pelvic veins for decades and are highly refractory to blood-borne immune defense elements. However, there is evidence that human populations can develop immune-mediated resistance to re-infection and, at least in animals, there is evidence that vaccine-related protective immune responses can be induced against the parasites. Schistosome larvae (cercariae) attenuated with gamma, X-ray, or ultraviolet radiation are capable of infecting their host through the skin but they do not survive to migrate beyond the lung stage; most are eliminated over a protracted time period of up to three weeks or more after infection (Harrop and Wilson, 1993). Laboratory animals immunized with radiation attenuated (RA) cercariae of *S*. *mansoni* and *S. haematobium* are protected against challenge infection with normal cercariae, with reductions in worm burdens compared to non-vaccinated animals varying from 30–90% depending on the host, schistosome species and strain, numbers of immunizations, and time of challenge following immunization (Dean, 1983). Its proven efficacy in primates (Eberl et al., 2001; Kariuki et al., 2004) has reinforced the validity of the RA model, and it is still the gold standard against which the protective efficacies of recombinant antigens are compared.

Although there is no doubt that the immune response elicited by RA-vaccination mediates parasite attrition, there is no firm consensus on the phenotype of the protective immune response. Initial studies determined that reduction in challenge worm burden in RA-immunized mice correlated with polarized Th1 immune responses, characterized by the production of interferon-gamma (IFN-γ) by leukocytes in the airways of the lung (Smythies et al., 1992; Wilson et al., 1996). However, significant protection in the RA model was also found in *IL-12p40*^-^^/^^-^mice (Anderson et al., 1998, 1999), and nitric oxide was shown not to be a major agent causing parasite elimination (Coulson et al., 1998). Furthermore, other studies have shown that effective immunity in the RA vaccine model involved Th2 (Mountford et al., 2001) or mixed Th1/Th2 (Hoffmann et al., 1999; Hewitson et al., 2005) immune responses. In contrast, studies on humans that exhibit resistance to infection following chemotherapy consistently indicate that Th2 immune responses correlate with protection (Ganley-Leal et al., 2006; Walter et al., 2006; McManus and Loukas, 2008; Jiz et al., 2009; Black et al., 2010a, b; Figueiredo et al., 2012; Fitzsimmons et al., 2012; Pinot de Moira et al., 2013; Wilson et al., 2014). It is possible that interpretation of these data is confounded by the role of T-cells in schistosome development. While the complete absence of CD4^+^ T-cells significantly impairs parasite growth and reproduction (Harrison and Doenhoff, 1983; Davies et al., 2001), so too does the suppression of Th2 immune responses during the pre-patent liver phase of infection (Riner et al., 2013). Both the lung and liver represent the major sites of attrition in RA-immunized mice challenged with *S. mansoni* (Laxer and Tuazon, 1992), suggesting that an alteration to the fine balance of Th1 and Th2 immune responses that occur in these anatomical sites at different times during natural infection may be sufficient to achieve protection, rather than a polarized response one way or the other.

What is clear is that irradiated cercariae elicit the production of IgG antibodies specific to parasite proteins, which induce protection when passively transferred to mice (Abath and Werkhauser, 1996). This correlates with the age-dependent development of human immunological resistance to reinfection with *S. mansoni*, which is also associated with the presence of anti-tegument IgG antibodies (Karanja et al., 2002). In addition, it has been proposed that the resistance to re-infection following chemotherapy with praziquantel, is mediated by antibodies specific to schistosome antigens released upon worm death that are not normally encountered by the host immune response (Mutapi et al., 1998; Gomes et al., 2002). These observations prompted searches to identify those molecules recognized by antibodies taken from RA cercariae-vaccinated mice, or humans resistant to re-infection. The most prominent of these were the tegument-associated antigens; Sm23 a member of the tetraspanin family of surface molecules (Da’dara et al., 2002, 2003), the fatty acid binding protein Sm14 (Tendler and Simpson, 2008) and the apical lipid bilayer-associated glucose transporter SGTP4 (Skelly et al., 1998; Krautz-Peterson et al., 2010). Despite the induction of parasite-specific cytokines and antibodies (Da’dara et al., 2002, 2003; Mahana, 2006), immunization with these antigens failed to elicit protection above the arbitrarily chosen 40% threshold (Bergquist and Colley, 1998). Moreover, recent studies of human immune responses to these candidates did not pin-point any with a particular potential as a vaccine (Ribeiro de Jesus et al., 2000; Al-Sherbiny et al., 2003).

It is likely that antigens on the surface membrane of schistosome parasites are inaccessible to antibody binding (Keating et al., 2006; Tallima and El Ridi, 2008) which ensures that the antigens are protected behind a tight, almost impermeable, sphingomyelin-based hydrogen-bond network (Tallima and El Ridi, 2008; Migliardo et al., 2014). On the other hand, molecules released by the parasite during migration and development (termed excretory-secretory products; ESP) readily interact with specific antibodies and other effectors of the host defense system. Because the lung was demonstrated as the major site of attrition of schistosomes after immunization with RA vaccines, the early developing schistosome larvae are considered vulnerable targets of innate and adaptive immunity (Dean, 1983; Coulson, 1997). We have suggested that effective immune responses directed against these ESP harm the juvenile parasites as they pass through the narrow and convoluted capillaries of the lung (El Ridi et al., 2010). Therefore, the ESP represents a potential pool of vaccine targets. A number of ESP of cercariae, *in vitro*-cultured and *ex vivo* lung-stage schistosomula, and adult worms of *S. mansoni* (Harrop et al., 1999; Knudsen et al., 2005; Curwen et al., 2006; Hansell et al., 2008; El Ridi and Tallima, 2009), *S. japonicum* (Liu et al., 2009; Liao et al., 2011) and *S. haematobium* (Young et al., 2012) have been identified; molecules common to these preparations include actin, heat shock proteins, enolase, aldolase, glutathione *S*-transferase, triose phosphate isomerase, glyceraldehyde 3-phosphate dehydrogenase (SG3PDH), 2 *cis*-peroxyredoxin (PRX), and serine and, predominantly, cysteine peptidases.

Schistosomes express several different classes of cysteine peptidases. *S. mansoni* cathepsin B1 (SmCB1), a member of the lysosomal cysteine peptidases of the papain superfamily, was found to be expressed at high levels in the caecum and protonephridia of cercariae. Expression increases in the parasite gut soon after skin penetration and schistosomular transformation corresponding to the initiation of blood feeding (Zerda et al., 1988). The peptidase was reported to be the major hemoglobin-digesting enzyme alongside another papain-like cysteine peptidase, cathepsin L1 (SmCL1), both of which are major proteins in worm soluble extracts and ESP (Day et al., 1995; Dalton et al., 1996; Caffrey et al., 1997; Brady et al., 1999a, b, 2000; Bogitsh et al., 2001). SmCL1 efficiently degrades human hemoglobin to absorbable dipeptides and amino acids and is localized to the gastrodermis and to the tegument of adult worms. It displays 44% identity at the amino acid level with a second cathepsin L (SmCL2) which, by contrast, is not detected in cercarial extracts or gut caecum, but is predominantly localized to the reproductive system of the female parasite and to the gynecophoric canal of the male, implying involvement with the worm reproductive physiology (Dalton et al., 1996; Brady et al., 1999a, b, 2000; Stack et al., 2011). More recently, a third cathepsin L member, SmCL3, was shown to be expressed in the worm gastrodermis and was found to hydrolyze hemoglobin and serum albumin (Dvorák et al., 2009). Furthermore, a second cathepsin B (SmCB2) was localized to the schistosome tegument and may be involved in its biogenesis (Wippersteg et al., 2005).

Cathepsin B and cathepsin L activities are readily detected by enzymatic assays in *S. mansoni* cercarial extracts and may facilitate skin penetration (Dalton et al., 1997; Kasný et al., 2007, 2009; Dvorák et al., 2008). 8–10 day old cultured larvae of *S. mansoni* also exhibited a dramatic increase in expression of these hemoglobin-degrading peptidases (Zerda et al., 1988; Dalton et al., 1995; Dalton and Brindley, 1996) and RNAi-mediated knockdown experiments showed that at least for SmCB1 the parasites cannot feed *in vitro* on hemoglobin and do not survive *in vivo* without this enzyme (Correnti et al., 2005). More recently, using microarray analysis, Gobert et al. (2010) showed that genes encoding cathepsin B and cathepsin L were greatly up-regulated in *S. mansoni* larvae cultured for 5 days (65 and 37-fold, respectively). Both SmCBs and SmCLs are highly immunogenic in infected mice and antibodies are detected in sera of *S. mansoni*-infected patients (Dalton et al., 1995; Dalton and Brindley, 1996; Planchart et al., 2007; Sulbarán et al., 2010).

The cathepsin B and L cysteine peptidases require the parasite gut-associated asparaginyl endopeptidase (Sm32) for their activation from an inactive zymogen to fully active mature enzyme (Dalton et al., 1995, 1996; Brindley et al., 1997; Skelly and Shoemaker, 2001; Sajid et al., 2003; Delcroix et al., 2006; Krautz-Peterson and Skelly, 2008; Stack et al., 2011). Sm32 is also a cysteine peptidase, but is not a member of the papain superfamily (Dalton et al., 1995; Dalton and Brindley, 1996). Given their central importance in the biology of the parasite *S. mansoni* cysteine peptidases have been of interest as both vaccine candidates for disease prophylaxis and potential chemotherapeutic targets (Dalton and Brindley, 1996; Wasilewski et al., 1996; Abdulla et al., 2007).

In addition to their role in the biology of the parasite, we have shown that helminth cysteine peptidases have an ability to modulate the host immune response (O’Neill et al., 2000; Donnelly et al., 2010). Cysteine peptidases from such diverse sources as papaya (papain; Sokol et al., 2008), house dust mite (Derp1; Roche et al., 1997; Kikuchi et al., 2006), *Leishmania mexicana* (Pollock et al., 2003), and many fungal allergens (Shen et al., 1998; Kheradmand et al., 2002) have all been shown to skew the immune response toward a Th2 phenotype characterized by the release of IL-33 and IL-4 and the production of antigen-specific IgG1. Despite differences in amino acid sequence and tertiary structure, their shared ability to induce Th2 immune responses is dependent on their common enzymatic activity. Such is the potency of their ability to modulate immune responses that at low doses these cysteine peptidases can act as adjuvants, inducing Th2 responses to bystander antigens in the absence of another adjuvant (Chapman et al., 2007; Cunningham et al., 2012). Indeed, we have shown that the subcutaneous administration of papain prior to a challenge infection with *S. mansoni*, switched the parasite-specific immune response toward a Th2 phenotype. Furthermore, this approach resulted in a significant level of protection (50%) from infection (El Ridi and Tallima, 2013).

The mechanism by which cysteine peptidase enzymes drive the amplification of Th2 immune responses is a subject of vigorous research. It has been suggested that proteases may degrade intracellular epithelia cell proteins, or damage epithelial cells, for example in the lung, which respond by secreting stress-related cytokines including IL-12, IL-33, and thymic stromal lymphopoietin (TSLP), that subsequently activate mast cells and basophils (Tang et al., 2010; Liang et al., 2012). Innate lymphocytes, termed lung natural helper (LNH) cells, which produce IL-33 and TSLP, have also been implicated in papain-induced airway inflammation (Halim et al., 2012). Recent studies have indicated that a primary function of TSLP, produced as a result of protease activity, is to prevent the induction of Th1-inducing molecules such as IL-12 and CD70 from innate immune cells (Massacand et al., 2009), which in turn facilitates the development of Th2 immune responses. Interestingly, we found that, like papain, a vaccine formulation containing a combination of TSLP or IL-33 and larval ESP molecules reproducibly elicited production of parasite-specific Th2 cytokines and antibodies in response to a challenge infection with *S. mansoni*. Similar to the result achieved using papain, this vaccine formulation also elicited a highly significant (*P* < 0.0001) reduction of 62 (TSLP) to 78% (IL-33) in worm burden and worm egg load in host liver and small intestine (El Ridi and Tallima, 2013).

Indirectly, papain has been shown to mediate the differentiation of macrophages toward a Th2-associated M2 phenotype and via direct interaction, papain-treated macrophages are more likely to differentiate into M2 cells after stimulation with bacterial lipopolysaccharide, a ligand that more commonly induces inflammatory, Th1-associated M1 macrophages (Nhu et al., 2010, 2012). Consistent with these observations, we have previously reported that macrophages isolated from mice given a single parenteral injection of either SmCB1 or a *Fasciola hepatica* cathepsin L1 (FhCL1) were inhibited in their ability to produce Th1-inducing cytokines (Donnelly et al., 2010). The parasite proteases inhibited the TLR-TRIF dependent mechanism of activation by Th1-associated ligands and thus we proposed that these macrophages were more permissive to stimulation with Th2-promoting ligands. In the absence of TLR3-dependent signaling, production of Th2 cytokines is significantly increased during a murine infection of *S. mansoni* (Joshi et al., 2008).

Therefore, we suggested that, like papain, SmCB1 could act as an adjuvant to elicit an antigen-specific Th2 immune response to co-injected parasite molecules. However, we also proposed that as immunization with SmCB1 would result in the production of cathepsin B-specific antibodies, it would likely elicit a higher level of protection than papain. To that end, SmCB1 was administered subcutaneously to outbred mice, which were then challenged with an infection of *S. mansoni* cercariae. Consistent with the data seen for papain and in agreement with our hypothesis, highly significant (*P* < 0.0001) and reproducible reduction of 50–70% in challenge worm burden was achieved in five consecutive experiments. Protection was associated with predominance of Th2-related cytokines and antibodies (El Ridi et al., 2014). However, we found that the reduction in worm egg counts in host liver and small intestine was not as striking as for the worm burden, in accordance with earlier findings correlating Th2 dominant responses with increased schistosome egg production (Wynn, 1999; Xu et al., 2009). Nevertheless, we observed that when a mixture of SmCB1 and FhCL1 were combined with the secreted proteins SG3PDH or SG3PDH and PRX-MAP a highly significant (*P* < 0.0001) and reproducible reduction of 66% in challenge worm burden and in worm egg load in liver and small intestine as well was achieved (El Ridi et al., 2014). The data confirmed that schistosome cysteine peptidases have in-built immune modulating properties that are protective on their own and have the potential to enhance the protective responses to other molecules. In line with all the evidence from allergen cysteine peptidases, this activity was related to their enzymatic activity since SmCB1 inactivated with inhibitors (E-64) or a non-active recombinant form of FhCL1 displayed markedly reduced level of Th2 mediators, and was associated with a significant decrease in protective capacity (El Ridi et al., 2014).

These results clearly demonstrate the induction of significant protection levels when an active parasite cysteine peptidase enzyme is used in the vaccine formulation with no additional requirement for an adjuvant. However, similar to the results achieved with the RA vaccination model, we have found inconsistencies between the characteristics of the antigen-specific T-cell response in protected animals. Instead, the key to the peptidase-mediated protective effect may be the stimulation of a particular type of antibody response. Mathematical models have concluded that in younger, untreated endemic human populations, parasite infections activate short-lived plasma cells that are essentially non-protective. In contrast, in older populations the cumulative deaths of infection worms, due to natural death or chemotherapy, releases an antigen load which stimulates a different immune response, characterized by the production of long-lived plasma cells which reduces worm load (Mitchell et al., 2012). While it is clear that cysteine proteases, irrespective of their source, clearly induce the production of Th2-type antibodies against themselves and bystander antigens, there has been no investigation into the nature of antibody-producing B-cell. Recently, it was reported that immunization of mice with low doses of a fish venom protease (Natterin), induced the differentiation of terminally differentiated, long-lived antibody-secreting cells and that this was dependent on the proteolytic activity of the natterin (Komegae et al., 2013). In addition, these authors demonstrated that the production of both IL-5 and IL-17 in response to the venom protease directly influenced the maintenance of the antibody secreting cells in the spleen (Grund et al., 2012). Therefore, its seems that the enzymatic activity of cysteine proteases, besides inducing antigen-specific cytokines, is also essential to generate survival signals necessary for the longevity of antibody secreting plasma cells.

Such potent, adjuvant-like effects of the cysteine peptidases may offer an innovative and feasible approach to developing a human vaccine formulation for protection against schistosomiasis. As we have learned, the delivery of parasite-secreted active cysteine peptidases alone or combined with other schistosome vaccine candidates, elicited levels of protection comparable to the bench-mark treatment of RA-cercariae. To date, the failure of many anti-schistosome vaccines has been attributed to the use of inappropriate adjuvants and/or delivery systems. Our data indicates that inclusion of active cysteine peptidases with in-built immunopotentiating activity in a vaccine preparation could preclude the need for a chemical adjuvant.

## Conflict of Interest Statement

The authors declare that the research was conducted in the absence of any commercial or financial relationships that could be construed as a potential conflict of interest.
